# Primary Stability of Kyphoplasty in Incomplete Vertebral Body Burst Fractures in Osteoporosis: A Biomechanical Investigation

**DOI:** 10.3390/bioengineering11080798

**Published:** 2024-08-07

**Authors:** Oliver Riesenbeck, Niklas Czarnowski, Michael Johannes Raschke, Simon Oeckenpöhler, René Hartensuer

**Affiliations:** 1 Department of Trauma, Hand and Reconstructive Surgery, University Hospital Münster, Albert-Schweitzer-Campus 1, Building W1, Waldeyerstraße 1, 48149 Münster, Germany; 2 Center for Orthopaedic, Traumatology, Handsurgery, and Sportsmedicine, Klinikum Aschaffenburg-Alzenau, 63739 Aschaffenburg, Germany

**Keywords:** kyphoplasty, incomplete burst fracture, biomechanics, primary stability, vertebral body, lumbar spine, spinal instability, spinal trauma, osteoporotic compression fracture

## Abstract

**Background:** The objective of our study was to biomechanically evaluate the use of kyphoplasty to stabilize post-traumatic segmental instability in incomplete burst fractures of the vertebrae. **Methods:** The study was performed on 14 osteoporotic spine postmortem samples (Th11–L3). First, acquisition of the native multisegmental kinematics in our robot-based spine tester with three-dimensional motion analysis was set as a baseline for each sample. Then, an incomplete burst fracture was generated in the vertebral body L1 with renewed kinematic testing. After subsequent kyphoplasty was performed on the fractured vertebral body, primary stability was examined again. **Results:** Initially, a significant increase in the range of motion after incomplete burst fracture generation in all three directions of motion (extension–flexion, lateral tilt, axial rotation) was detected as proof of post-traumatic instability. There were no significant changes to the native state in the adjacent segments. Radiologically, a significant loss of height in the fractured vertebral body was also shown. Traumatic instability was significantly reduced by kyphoplasty. However, native kinematics were not restored. **Conclusions:** Although post-traumatic segmental instability was significantly reduced by kyphoplasty in our in vitro model, native kinematics could not be reconstructed, and significant instability remained.

## 1. Introduction

Kyphoplasty was introduced in 1998 by Mark Reiley [1] and has become widely accepted as a treatment of osteoporotic vertebral compression fractures (OCFs) [2,3,4]. Kyphoplasty offers advantages over vertebroplasty for restoring vertebral body height and kyphosis angle. Although its short-term effects, especially regarding pain relief, seem to be similar to those of vertebroplasty, kyphoplasty may have advantages in safety and long-term effects in OCFs [2].

The safety and successful outcomes of kyphoplasty have led to more liberal indications for the procedure. When kyphoplasty was first introduced, involvement of the middle column of the vertebral body was considered to be a contraindication for the procedure [3]. However, today it is considered safe to treat these fractures with cement augmentation techniques [5,6,7]. Some researchers have proposed that kyphoplasty is safe and effective as a stand-alone treatment even for burst fractures, but no studies have provided strong evidence for this [8].

However, biomechanical investigations have confirmed that cement can stabilize osteoporotic vertebrae under cyclic loading (axial compression). Therefore, vertebral augmentation techniques such as vertebroplasty and kyphoplasty are considered to be effective and minimally invasive surgical methods for the stabilization of fractured vertebrae [9]. These biomechanical results, restoring vertebral resistance to compression forces, might partially explain the reported success of kyphoplasty in pure compression fractures. Correction of kyphosis, vertebral body height, and resistance against compression addresses the main pathology mechanism. In traumatic incomplete burst fractures, additional injuries to the disc and ligaments may influence the stability of the motion segment, the functional spinal unit (FSU).

Previous studies have shown that augmentation without correction of the compressed fracture (vertebroplasty) did not restore the stability of the FSU in a human cadaveric incomplete burst fracture model [10].

Considering the stabilization of wedge-compression fractures—A1 according to AO spine classification—by kyphoplasty in a multisegmental, biomechanical model, contradictive results have been reported [11]. Disch and Schmoelz showed that vertebral body height can be restored, but they found that kyphoplasty could not restore the stability of an intact segment. They found that the initial gain in stability after kyphoplasty was markedly reduced to the level of the fractured specimen with increased cyclic load [12]. Achatz et al. reported that kyphoplasty was neither able to restore the initial vertebral body height, nor could it restore the kinematics of the intact spinal segment, which deteriorated under further cyclic loading [13]. Contrary to this, Holyoak et al. reported that kyphoplasty was able to restore vertebral body height close to the intact status and subsequent cyclic loading did not deteriorate height relevantly [14]. To our knowledge, reports of only two additional studies of the biomechanics of kyphoplasty for complete burst fractures—A4 according to AO spine classification—are available: Wong et al. reported that kyphoplasty failed to sufficiently restore stability as a stand-alone treatment after high-energy burst fracture [15]. Germaneau et al. concluded that percutaneous kyphoplasty offers good primary stability in burst fractures, but that its success is limited by potential lesions in adjacent discs or ligaments [16].

Because, to the best of our knowledge, no study has presented information on the stabilization of incomplete burst fractures—A3 according to the AO spine classification and OF 3 according to the OF classification—by kyphoplasty, we conducted a human cadaveric study using a robot-based spine tester and performed three-dimensional motion analysis [11,17]. The spine tester has previously been evaluated for single and multilevel testing [18].

We hypothesized that kyphoplasty can restore primary stability in a traumatic incomplete burst fracture model.

## 2. Materials and Methods

### 2.1. Specimens

We used 13 human fresh-frozen cadaveric spine samples (Th11–L3). The median age of the specimen donors was 82 years (Q1 [first quartile] = 75 years; Q3 [third quartile] = 83 years), and all donors were female. In all samples, bone mineral density (BMD) was measured using quantitative computed tomography. The median BMD was 75.63 mg/cm^3^ (Q1 = 70.32 mg/cm^3^; Q3 = 91.18 mg/cm^3^). In comparison, a BMD of >120 mg/cm^3^ is considered normal, one between 80 and 120 mg/cm^3^ indicates osteopenia, and one of <80 mg/cm^3^ indicates osteoporosis [19]. Therefore, all samples except one were from donors who had osteopenia or osteoporosis. Samples with relevant morphologic changes beyond age-related degeneration (e.g., tumor, fracture, deformity, fusion) were excluded.

Prior to testing, all specimens were thawed slowly to room temperature and all soft tissue and muscles were dissected carefully to preserve osseous and ligamentous structures.

The caudal and cranial vertebral bodies were rigidly fixed in a standardized manner in a custom-made embedding frame filled with a two-component resin (Technovit 3040, Heraeus Kulzer GmbH, Hanau, Germany). This setup was then attached to customized tools to mount the samples into the servo-hydraulic testing machine and the testing robot. All samples were kept moist during the dissection and testing processes, and the whole procedure was performed in accordance with the process outlined by Wilke et al. [20].

### 2.2. Fracture Creation

**Figure 1:**
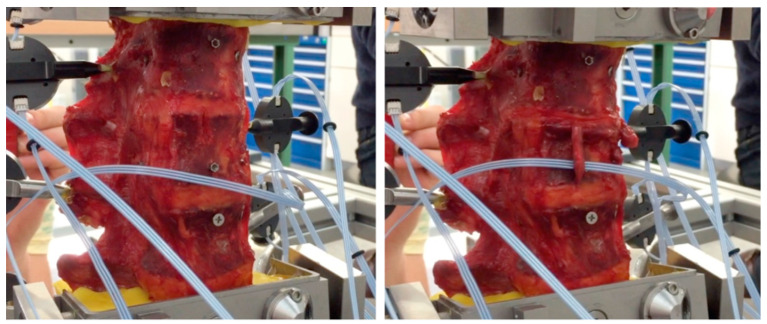
Modified fracture creation by distance-controlled compression after osteotomy-like weakening of the upper endplate L1. Specimen before compression (**left**) and after fracture induction by axial compression (**right**).

**Figure 2:**
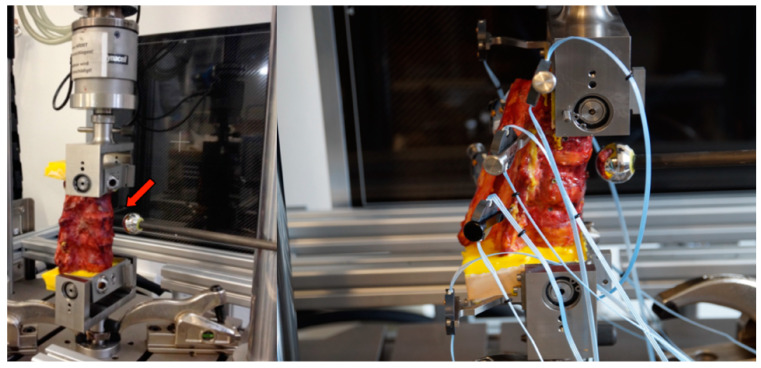
Hydraulic material testing machine used to create standardized incomplete burst fractures and obtain radiographs. At the **left**, the mounted motion capture marker (rigid bodies) and radiographic reference (arrow) are shown. At the **right** is a magnified lateral view of a mounted sample.

We used a previously validated and reported protocol for the fracture creation, adding a novel mounting frame in the servo-hydraulic testing machine and in the robot for kinematic testing (Figure 1 and Figure 2) [21].

The combination of an osteotomy and a distance-controlled compression using a hydraulic testing machine (Instron 8874, Instron, Norwood, MA, USA) resulted in the reproducible creation of type A3 incomplete burst fractures according to the AO spine classification and an OF 3 according to the OF classification [11,17].

### 2.3. Kyphoplasty

**Figure 3:**
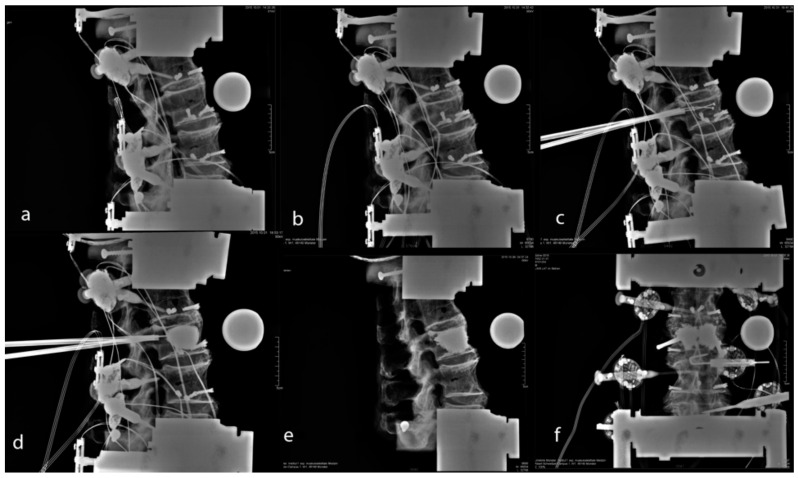
Radiographs of the native sample (**a**), fractured specimen (**b**), balloon in position (**c**), inflated balloon (**d**), inserted cement (**e**), and after cement insertion using the anteroposterior technique (**f**).

Kyphoplasty (Figure 3) was performed by a single experienced spine surgeon (RH) who followed the manufacturer’s recommendations. To simulate permanent pressure even in the supine position in vitro, a constant compressive pressure of 100 N was applied during balloon inflation, and balloon pressure was recorded. After the balloon was fully inflated, the position of the hydraulic testing machine was then preserved. After balloon deflation, there was no compressive pressure in the upright testing setup. Polymethylmethacrylate was loaded into the vertebral body according to the manufacturer’s recommendations. The amount of polymethylmethacrylate was assessed via a lateral radiograph in imitation of clinical practice, and the volume of cement intrusion was recorded.

### 2.4. Kinematic Testing

**Figure 4:**
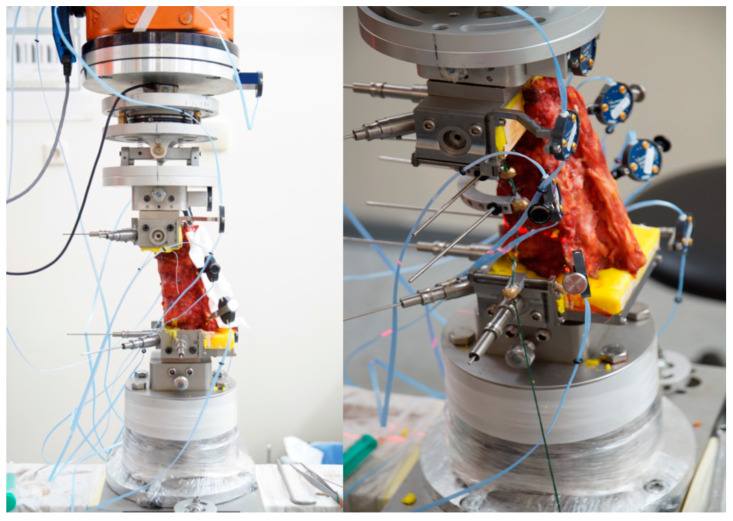
Mounted specimen in the robot-based spine tester combined with active optical motion tracking to record each single segmental kinematic behavior in a multisegmental setup: overview (**left**) and magnified view with rigid bodies and follower-load applications (**right**).

A first set of kinematic tests was conducted with the intact specimens, both with and without follower load, using a robot-based system combined with a custom-made cardan drive that ensured the application of pure moments (7.5 Nm) for extension–flexion, lateral flexion, and axial rotation (Figure 4) [22,23]. All further tests were performed under follower load conditions (350 N).

Intersegmental movement was additionally recorded by the optical motion tracking system to evaluate the kinematic behavior of each FSU in the multisegmental test setup.

After fracture creation and after kyphoplasty, kinematic testing was repeated to compare the individual effects for each specimen.

### 2.5. Radiological Assessment

Reconstruction of the vertebral body was monitored by calibrated radiographic examinations, in accordance with clinical practice.

**Figure 5:**
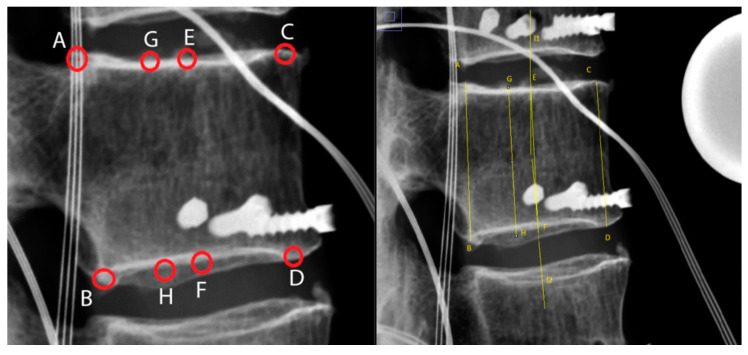
Schematic presentation of height measurement via lateral radiographic projection: posterior (AB), anterior (CD), middle (EF), and posterior two-thirds (GH). I1 and I2 are perpendicular midline intersections for the construction of the points E and F [24].

Height for intact vertebral bodies, fractured bodies, and reconstructed bodies was monitored using lateral radiographs (Figure 5). Qualitative monitoring of height restoration was performed by modifying the method described by McKiernan et al. [24]. Measurements obtained included posterior vertebral body height (AB), as shown on lateral radiographs; anterior vertebral body height (CD); central height (height of the middle of the vertebral body (EF)); and the height between the posterior one-third and the anterior two-thirds of the vertebral body (GH).

### 2.6. Groups

Each specimen was tested intact without follower load (group 1), intact with follower load (group 2), fractured (group 3), and after kyphoplasty (group 4).

### 2.7. Statistics

Statistical analysis was performed using the Wilcoxon signed-rank test and Bonferroni correction using SPSS (SPSS^®^ Statistics 27; IBM, Endicott, NY, USA).

## 3. Results

### 3.1. Fracture Creation and Vertebral Body Reconstruction

The vertebral body height was decreased by the standardized fracture creation procedure to the following percentages of intact values: AB, 91.9%; CD, 75.1%; EF, 76.6%; and GH, 76.6%.

The median balloon inflation pressure was 13.5 bar (Q1 = 12; Q3 = 14.25). The median balloon volume was 10 mL (Q1 = 9; Q3 = 12.5). The median cement volume was 9.6 mL (Q1 = 9; Q3 = 12).

**Figure 6:**
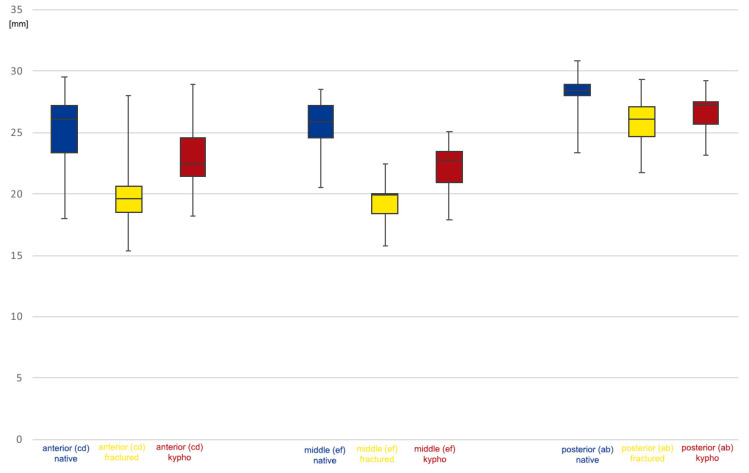
Boxplot of lateral vertebral body height in millimeters. Anterior (cd), middle (ef), and posterior (ab) values are presented by condition: intact, blue; fractured, yellow; reconstructed by kyphoplasty (kypho), red.

We were able to reconstruct the vertebral body height, using balloon kyphoplasty, to the following percentages of intact values: posterior (AB), 95.8%; anterior (CD), 86.2%; middle (EF), 88%; and posterior two-thirds (GH), 85.9%. Table 1 provides details of the losses of height after fracture creation and after kyphoplasty; Figure 6 provides an overview of the lateral vertebral body heights.

### 3.2. Kinematics of the Injured Segment (Th12–L1)

An increase in the range of motion (ROM), in the size of the neutral zone, and in the size of the elastic zone after fracture induction was obvious for axial rotation, extension–flexion, and lateral flexion.

**Figure 7:**
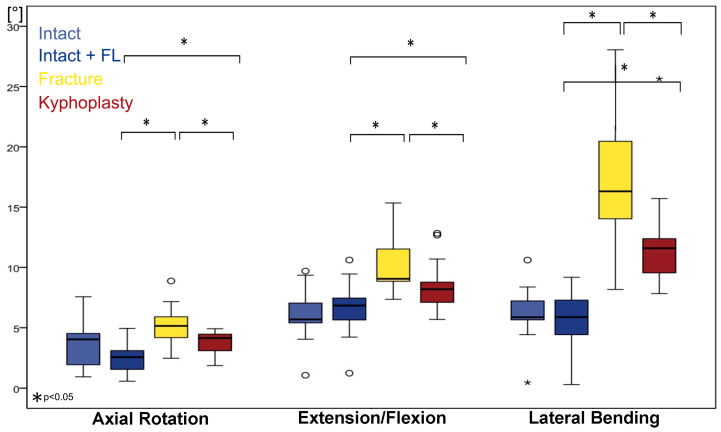
Boxplot of kinematic median values of functional spinal unit Th12–L1 for axial rotation, extension–flexion, and lateral flexion. Intact values without (light blue) and with follower load (blue), fractured values with follower load (yellow), and values after kyphoplasty with follower load (red). Circle represents outliers and five-pointed asterisk represents extreme outliers.

**Figure 8:**
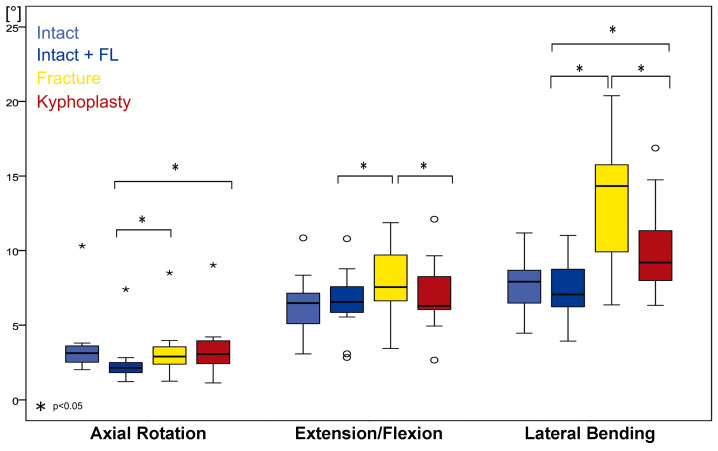
Boxplot of kinematic median values of functional spinal unit L1–L2 for axial rotation, extension–flexion, and lateral flexion. Intact values without (light blue) and with follower load (blue), values for fractures with follower load (yellow), and values after kyphoplasty with follower load (red). Circle represents outliers and five-pointed asterisk represents extreme outliers.

In Figure 7 and Figure 8, we considered the intact condition with (light blue) and without follower load (blue) and the fractured condition (yellow) as reference points for estimating the effect of kyphoplasty (red).

### 3.3. Extension–Flexion

In extension–flexion in the intact condition, ROM without follower load was 5.7° (Q1 = 5.2°; Q3 = 8.0°); under follower load, it was 6.8° (Q1 = 5.4°; Q3 = 8.4°). ROM increased after fracture by 132% (*p* < 0.05) to 9.1° (Q1 = 8.8°; Q3 = 12.4°). This change can be interpreted as traumatic segmental instability. After kyphoplasty, ROM decreased by 90% (*p* < 0.05). However, in comparison with the intact state, a significant increase of 120% (*p* < 0.05) still remained.

### 3.4. Axial Rotation

Axial rotation in the intact condition without follower load was 4.0° (Q1 = 1.8°; Q3 = 4.6°); under follower load, it was 2.6° (Q1 = 1.2°; Q3 = 3.2°). ROM increased after fracture to 5.1° (150%; *p* < 0.05; Q1 = 3.7; Q3 = 6.1°). These changes can be interpreted as traumatic segmental instability for rotation. After kyphoplasty, ROM significantly decreased to 4.1° (Q1 = 2.6°; Q3 = 4.5°; *p* < 0.05). However, in comparison with the intact state, a significant increase in ROM in axial rotation after kyphoplasty remained: 161% (*p* < 0.05).

### 3.5. Lateral Flexion

In lateral flexion, intact ROM without follower load was 5.9° (Q1 = 5.2°; Q3 = 7.8°); under follower load, it was 5.9° (Q1 = 4.0°; Q3 = 7.4°). ROM increased after fracture by 277% (*p* < 0.05) to 16.3° (Q1 = 13.9; Q3 = 20.7°). These changes can also be interpreted as traumatic segmental instability for lateral flexion. After kyphoplasty, ROM decreased to 11.6° (Q1 = 9.5°; Q3 = 12.4°; *p* < 0.05). However, in comparison with the intact state, a significant increase in ROM (197%; *p* < 0.05) remained.

### 3.6. Kinematics of L1–L2

The kinematics of levels L1–L2 show effects similar to those at the experimentally injured level. This effect of partial restoration of segmental stability was detectable for all movement directions: axial rotation, extension–flexion, and lateral flexion (Figure 8; Table 2).

### 3.7. Adjacent Segments

Each segment was evaluated independently using optical three-dimensional motion analysis. No significant changes were detected in the segments except FSUs involving fractured vertebra (Th12–L1 and L1–L2).

## 4. Discussion

In the common understanding of spinal instability, (incomplete) burst fractures are considered to be unstable. However, clinical treatment options do not necessarily reflect this assessment under the current understanding of biomechanics.

Previous studies have shown that vertebroplasty without reconstruction of vertebral body height could not restore the stability of the FSU in a human cadaveric burst fracture model [10].

These findings must be discussed within the framework of conflicting clinical findings regarding treatment success with cement augmentation. According to Germaneau et al., kyphoplasty can stabilize a traumatic fractured segment [16]. Therefore, they concluded that percutaneous kyphoplasty offers sufficient primary stability in burst fractures.

This is consistent with some other case reports noting that kyphoplasty should be a reliable and successful stand-alone option for treating traumatic burst fractures [8]. However, Wong et al. reported that kyphoplasty failed to sufficiently restore stability as a stand-alone treatment after high-energy burst fracture due to the compromised intervertebral discs [15]. Their biomechanical results are supported by multiple clinical reports, including those of Oner et al. [25], Zaryanov et al. [26], Josten et al. [27], and Spiegl et al. [28] of the need to use both posterior instrumentation and kyphoplasty to achieve vertebral body restoration and segmental stabilization.

Our findings add to the controversy by showing that kyphoplasty has some potential to increase segmental stability in a traumatic incomplete burst fracture model. We found that the increase in stability in extension/flexion, rotation, and bending was significant compared with that in the fractured state. This effect was evident in the injured index level (Th12–L1) as well as in the level below (L1–L2). It seems obvious that the reconstruction of height leads to some stabilizing effects in both involved FSUs. This effect might be in accordance with the flagpole principle described by Evans [29].

However, the post-surgery values we obtained did not reach the values of intact kinematic conditions, and significant segmental instability remained compared with the intact sample. Therefore, our findings show that kyphoplasty cannot reconstruct native kinematic values after incomplete burst fracture.

In fact, there is little knowledge about the resulting instability in vivo after incomplete burst fractures. This lack of knowledge is replenished by different treatment options, including conservative [30,31], vertebroplasty [32], kyphoplasty [33], instrumentation [30], different combinations of kyphoplasty [31], and instrumentation plus 360° fusion [30].

One explanation may be an inconsistent usage of the term incomplete burst fracture. This fracture type has a wide range of appearances that can lead to different levels of instability. For that reason, we advocate for the usage of differentiated classification systems to standardize the type of injury in clinical practice and experimental research. Even when using a specific classification for osteoporotic vertebral fractures and a corresponding scoring system, it remains difficult to recommend a treatment method. Therefore, additional posterior instrumentation should be evaluated in the presence of an OF 3 fracture [34,35].

Additionally, the importance of active stabilization of the FSU must be discussed. It is well known that biomechanical kinematic studies mainly investigate the passive factors of motion. Some may simulate muscle forces and have shown a glimmer of importance [36]. However, post mortem experiments cannot verify the role of the active motion system in stabilizing the spine, resulting in a limitation of our study.

Van Dieen et al. reported that the changed trunk muscle recruitment patterns in patients with low back pain enhance the stability of the lumbar spine [37]. These findings indicate the potential compensatory mechanisms of the active motion system in case of a resulting segmental instability and may partially explain why some patients gain enough passive stability to compensate after kyphoplasty and others do not. As long as we do not have the tools to evaluate the patient’s active compensatory potential and calculate the required passive stability, we must rely on the findings of kinematic post mortem studies.

Another limitation of our study is the volume of cement used. The cement volumes in the published literature vary significantly, but smaller amounts of cement may be used in everyday clinical practice [14,38,39]. Therefore, our study might overestimate the effect of biomechanical stabilization by kyphoplasty in incomplete burst fractures.

## 5. Conclusions

Kyphoplasty is able to stabilize incomplete burst fractures by restoring vertebral body height, but significant instability remains in comparison with intact values. Therefore, successful treatment depends not only on correct execution of the procedure, but also on the individual capacity of active segmental stabilization to some extent.

## Figures and Tables

**Table 1:** Vertebral body height (millimeters) after fracture creation and after kyphoplasty.

Specimen	1	2	3	4	5	6	7	8	9	10	11	12	13	Median	Q1	Q3
**Native**																
Anterior (CD)	22.5	26.4	27.5	25.8	20.7	25.1	27	26.1	24.3	27.8	26.8	28.4	22.1	26.1	23.4	27.3
Middle (EF)	21.8	26.3	25.3	27	24.4	25.7	26.3	24.8	26.5	26.3	27.2	25.9	24.3	25.9	24.6	26.4
Posterior two-thirds (GH)	23	27	25.6	27.4	24.9	26.5	27.4	24.2	27.2	27.1	27.2	26.9	24.9	26.9	24.9	27.2
Posterior (AB)	28.8	28.8	28.3	28.3	23.8	28.6	29	27.9	28.1	28.4	29.1	30.3	26.9	28.4	28	28.9
**Fractured**																
Anterior (CD)	18.8	16.5	27	21.9	21.2	19.8	18.3	19.1	18.7	16.9	19.9	19.6	20	19.6	18.5	20.6
Middle (EF)	18.3	17.3	22.4	22	17.6	21.5	18.5	18.6	21	19.1	20.9	19.9	20.6	19.9	18.4	21.3
Posterior two-thirds (GH)	19.9	19	22.9	22.1	19.3	22.4	20	18.5	21.9	20.6	21.2	22	22.3	21.2	19.6	22.2
Posterior (AB)	27.1	26.1	26	24.9	24.5	26.6	23.9	23.2	27.2	24.8	27.1	28.3	26.5	26.1	24.7	27.1
**Reconstructed**																
Anterior (CD)	22.5	19.2	26.8	23.2	21.9	21	23.5	22.5	20.4	22.6	26	25.7	22	22.5	21.5	24.6
Middle (EF)	21	19.7	23.2	24.4	20.9	22.8	19.7	21.1	21.9	23.2	23.5	23.8	23.5	22.8	21.0	23.5
Posterior two-thirds (GH)	23.1	21.2	23.3	24.3	21.9	23.5	20.5	20.7	22.3	23.2	23.4	25.1	23.5	23.2	21.6	23.5
Posterior (AB)	28.6	26	27.2	27.6	24.8	27.2	24.7	25.3	27.1	26.8	27.5	28.9	27.4	27.2	25.7	27.6

AB, posterior vertebral body height; CD, anterior vertebral body height; EF, central vertebral body height, aka height of the middle of the vertebral body; GH, height between the posterior one-third and the anterior two-thirds of the vertebral body; Q1 and Q3, median values for first and third quartiles, respectively.

**Table 2:** Kinematics (range of motion [°]), Q1 = 1st quartile; Q3 = 3rd quartile.

	Group 1: Intact without Follower Load			Group 2: Intact with Follower Load			Group 3: Fracture with Follower Load			Group 4: Kyphoplasty with Follower Load		
	Median	Q1	Q3	Median	Q1	Q3	Median	Q1	Q3	Median	Q1	Q3
Axial rotation												
Th11–Th12	5.2	2.0	5.7	3.3	1.7	4.6	3.4	1.8	4.5	3.1	1.8	5.0
Th12–L1	4.0	1.8	4.7	2.6	1.2	3.2	5.1	3.7	6.1	4.1	2.6	4.5
L1–L2	3.1	2.5	3.8	2.1	1.8	2.6	2.9	2.1	3.6	3.1	2.1	4.1
L2–L3	4.4	3.4	6.1	2.6	2.2	4.2	2.7	2.4	4.2	2.9	2.4	4.3
Extension-Flexion												
Th11–Th12	4.8	3.8	5.6	4.6	3.5	5.6	4.7	4.2	6.6	5.0	3.0	6.7
Th12–L1	5.7	5.2	8.05	6.8	5.4	8.4	9.1	8.8	12.4	8.2	6.8	9.7
L1–L2	6.5	5.1	7.2	6.5	5.7	7.8	7.5	6.0	10.0	6.3	5.5	8.9
L2–L3	8.2	6.3	8.9	8.6	6.3	9.7	9.0	6.2	9.9	9.6	6.3	9.9
Lateral flexion												
Th11–Th12	4.2	3.5	6.4	3.1	1.7	4.5	3.6	1.8	4.6	3.8	1.4	4.7
Th12–L1	5.9	5.2	7.8	5.9	4.0	7.4	16.3	13.9	20.7	11.6	9.3	14.0
L1–L2	7.9	6.3	8.9	7.1	6.1	8.9	14.3	9.2	16.0	9.2	7.4	12.4
L2– L3	9.9	6.7	11.7	9.0	5.9	11.2	9.8	5.9	14.9	9.6	6.6	13.2

## Data Availability

Dataset available on request from the authors.
